# The Effects of Dietary Fishmeal Substitution by Full-Fat and Defatted *Zophobas morio* Larvae Meals on Juvenile Gilthead Seabream (*Sparus aurata*): An Integrative Approach

**DOI:** 10.1155/anu/8885509

**Published:** 2025-05-25

**Authors:** Adamantia Asimaki, Pier Psofakis, Elli-Zafeiria Gkalogianni, Aikaterini-Maria Katouni, Panagiotis Berillis, Konstantinos A. Kormas, Christos I. Rumbos, Christos G. Athanassiou, Antigoni Vasilaki, Eleni Fountoulaki, Morgane Henry, Eleni Mente, Enric Gisbert, Ioannis T. Karapanagiotidis

**Affiliations:** ^1^Aquaculture Laboratory, Department of Ichthyology and Aquatic Environment, Faculty of Agricultural Sciences, University of Thessaly, Volos, Greece; ^2^Laboratory of Entomology and Agricultural Zoology, Department of Agriculture, Crop Production and Rural Environment, Faculty of Agricultural Sciences, University of Thessaly, Volos, Greece; ^3^Department of Agriculture, University of Patras, Messolonghi, Greece; ^4^Institute of Marine Biology, Biotechnology and Aquaculture, Hellenic Centre for Marine Research, Anavyssos, Greece; ^5^Laboratory of Ichthyology, Culture and Pathology of Aquatic Animals, School of Veterinary Medicine, Aristotle University of Thessaloniki, Thessaloniki, Greece; ^6^Aquaculture Program, Institute of Agrifood Research and Technology, IRTA, la Ràpita, Spain

**Keywords:** digestive enzymes, fatty acid, histology, insect meal, intestinal microbiota, proximate composition, sustainable aquafeeds

## Abstract

This study evaluated the dietary fishmeal substitution by full-fat (FF) and defatted (DF) *Zophobas morio* meals regarding growth, feed efficiency, proximate and fatty acid compositions, digestive enzymes activities, histology and midgut microbiota in gilthead seabream (*Sparus aurata*). Juveniles initially weighing 3.4 g were distributed to triplicate groups and fed at satiation six isonitrogenous (8.41%) and isocaloric (21 Mj/kg) diets for 100 days. An insect meal-free diet was the control (CTRL), two diets contained a FF *Z. morio* meal at 49 g/kg (FF-49) and 97 g/kg (FF-97), and three diets contained a DF *Z. morio* meal at 58 g/kg (DF-58), 116 g/kg (DF-116) and 174 g/kg (DF-174) at the expense of fishmeal. Neither the form nor the inclusion level of *Z. morio* meals affected the feed intake of fish denoting a similar acceptability to that of fishmeal. Fish survival, growth and feed efficiency were not impaired by all dietary inclusion levels of *Z. morio* meals. Proximate composition of fish was altered but without indicating a clear correlation with the form or inclusion level of *Z. morio*. Increasing inclusions of both forms of *Z. morio* meals tended to decrease 22:6n-3, 20:5n-3, 18:3n-3 and 18:2n-6 levels in fish tissues. All fish exhibited similar proteolytic enzyme activities, but the increasing inclusions of both insect meal forms led to gradual increases in the lipase and α-amylase activities indicating a compensatory mechanism for lipid and carbohydrate digestion. The use of *Z. morio* meals led to some mild histomorphological changes in the intestine and liver that were more pronounced in fish fed the FF form at the highest inclusion level. Midgut bacterial communities of the groups were similar and dominated by potentially beneficial members of *Saccharimonadales* and *Rhodobacteraceae*, except FF-97 fish that had high abundances of *Legionella*- and *Pandoraea*-like bacteria. To conclude, *Z. morio* meal, either FF or DF, is a suitable insect protein for fishmeal substitution towards more sustainable aquafeeds for *S. aurata*.

## 1. Introduction

Sustainable aquaculture very much relies on sustainable aquafeeds that make a limited use of wild-sourced fishmeals and an increased use of feedstuffs that are produced with a lower environmental impact. After their authorisation for use in European aquafeeds (EC 893/2017), insect meals have become the subject of an extensive and intensified research [1, 2] assessing their suitability for fishmeal replacement in the majority of farmed fish species' diet [3–7]. This is because insect meals could serve both as high-quality proteins and sustainable feedstuffs. They are rich in protein [3, 8, 9] that is highly digestible by fish and in some cases comparable to that of fishmeal [5, 10], while certain insect species are good sources of lysine or methionine [8, 11–13] that are typically the limiting amino acids in low-fishmeal fish feeds. Furthermore, an increasing amount of research have also revealed their immunostimulating properties, which are mainly attributed to their content in antimicrobial peptides, lauric acid and acidic or nondigestible polysaccharides like silkrose, chitin and chitosan, so that insect meals could play significant roles in fish health enhancement and disease resistance [14–16].

The environmental impact of the use of insect meals in aquafeeds still needs a further and broaden understanding as there is a range of approaches and features that are involved [1, 17–19], while this impact can also be species-specific to the insect and farmed fish in concern [20–22]. The environmental benefits of the insect meal production have been documented and mainly involve the limited arable land and water, the highly efficient feed conversion and the low greenhouse gas emissions [17–19, 23]. Also, their high efficiency in converting food side streams such as fruit and vegetable by-products into insect biomass contributes to a circular economy approach that match several sustainable development goals [24, 25]. On the other hand, a greater energy use than that of conventional protein sources has been linked with insect production systems [18, 19]. Thus, it has been stressed that several modifications to the insect farming processes should be adopted to reduce the environmental consequences of insect meals such as facilities that use less energy combined with nutritional balanced diets that use organic side-streams for farmed insect nutrition [1, 19, 26]. Besides, an increased market availability, through production upscaling, lower trading prices and a stable quality of insect meals are needed [10, 27] for the aquafeed industry to make full use of these environmentally friendlier alternatives to fishmeal.

Among the insect meals used so far for fishmeal substitution in aquafeeds, those of *Hermetia illucens* and *Tenebrio molitor*, are the most studied ones [6, 19] probably due to their easy mass rearing production and worldwide availability [28, 29]. However, there are other species that could potentially serve as dietary fishmeal alternatives. The giant mealworm or superworm (*Zophobas morio*, Tenebrionidae), yet not listed in EC Regulation 893/2017, has recently been the focus of studies assessing its suitability as an alternative ingredient for livestock animal and fish feeds [30]. The species can be successfully reared on several amylaceous feedstuffs, while it also has the ability to utilise organic side-streams and by-products [30–32]. Except for the high energy use when cultured in cold climate countries, the environmental impact of its production is lower in comparison with livestock animal proteins [23]. The nutritional value of *Z. morio* larvae has been cited in several review studies [11, 30, 33, 34], indicating a high nitrogen/protein content that is similar to that of other insect species with substantial levels of all essential amino acids except for a poorness in methionine typical to most insect meals. *Z. morio* is known to deposit very high levels of fat during its larval stage, usually ranging 38%–42% of its dry matter [9, 11, 30, 35–38]. As a typical insect fat, the lipids of *Z. morio* are characterised by high levels of saturated fatty acids (SFAs) and traces of the valuable n-3 highly unsaturated fatty acids (HUFAs) [39], so that its inclusion in diets may pose the risk of deteriorating the lipid nutritional value of fish. Therefore, the defattening of *Z. morio* meal may be a better option for the nutrition of marine fish species that have high requirements for n-3 HUFA and a relatively low SFA content in their tissues. Besides, the defattening of insect meals raises the protein content of the end-product, offers a better stability against lipid oxidation and may yield a better fish growth [3, 10, 40].

To date, various studies have assessed the effects of fishmeal substitution by dietary *Z. morio* meal in fish and crustacean diets. These have been conducted in species such as Nile tilapia (*Oreochromis niloticus*) [41–45], cobia (*Rachycentron canadum*) [46], rainbow trout (*Oncorhynchus mykiss*) [47, 48], European seabass (*Dicentrarchus labrax*) [49, 50], Asian seabass (*Lates calcarifer*) [51], sea trout (*Salmo trutta m. trutta*) [52], perch (*Perca fluviatilis*) [53] and Pacific white shrimp (*Penaeus vannamei*) [54]. In gilthead seabream (*Sparus aurata*), Mastoraki et al. [50] assessed the nutrient digestibility of a diet containing *Z. morio* meal, while Henry et al. [15] reported its immunomodulatory effects. Our knowledge, however, of an integrative approach of fishmeal substitution by *Z. morio* meal on important performance indicators such as the feed intake, growth performance, feed utilisation, tissue nutrient deposition, fatty acid profiles, digestive enzymes, histomorphology and intestinal microbiota of *S. aurata* is still lacking. The species is the most significant in terms of annual biomass production (344,393 mt) and the second in terms of economic importance (value USD 2.01 billion) among Mediterranean farmed fish [55], and successful dietary modifications implementing insect meals to lower the wild-sourced fishmeal inclusion levels can improve the environmental sustainability of its intensive farming.

## 2. Materials and Methods

### 2.1. Ethics Statement

The aquaculture facilities of the Laboratory of Aquaculture (University of Thessaly) are licenced (EL-43BIO/exp-01) by the national authorities to operate trials with the use of laboratory animals under the guidelines of the Directive 2010/63/EU. The research protocol was authorised (2019/16048) by the University's Ethical Committee, and the experimental procedures were performed in accordance with the ARRIVE guidelines by scientists accredited by FELASA.

### 2.2. Insect Meal and Experimental Diets

*Z. morio* larvae were cultured at the University of Thessaly (Laboratory of Entomology and Agricultural Zoology) feeding on wheat bran (bought from a local retailer) and a commercial poultry feed (No. 3, Compound Feed for Layers, Viozokat S.A., Katerini, Greece) at a ratio of 9:1 [30]. The larvae were milled and dehydrated at 60°C for 12 h to get a full-fat (FF) *Z. morio* larvae meal with 31.3% protein and 39.7% lipid content (Table 1). A batch of the *Z. morio* larvae meal was then defatted (DF) using ether with continuous mixing and warming at 40°C for 1 h, and the procedure was performed twice to maximise fat removal. The solvent was then evaporated under a fume cupboard for 24 h and the DF *Z. morio* larvae meal contained 53% protein and 3.8% lipid (Table 1).

Six diets were formulated to be isonitrogenous (8.41% as fed) and isoenergetic (21.1 Mj/kg) (Table 2) satisfying the minimum amino acid and fatty acid requirements (Table 3) of *S*. *aurata*. The fishmeal used in the experimental diets contained 65.5% protein and 10.1% lipid (Table 1). The control (CTRL) diet contained 613 g/kg of fishmeal and no insect meals. Two diets were formulated to include FF *Z. morio* meal replacing fishmeal protein of the CTRL diet at 5% and 10%, thus corresponding to a dietary inclusion at 49 g/kg (diet FF-49) and 97 g/kg (diet FF-97), respectively. Another three diets were formulated to include the DF *Z. morio* meal replacing fishmeal protein of the CTRL diet at 10%, 20% and 30%, thus corresponding to a dietary inclusion at 58 g/kg (diet DF-58), 116 g/kg (diet DF-116) and 174 g/kg (diet DF-174), respectively. All diets were formulated to be isonitrogenous (8.41% as fed) and isoenergetic (21.1 Mj/kg) satisfying the minimum amino acid requirements of *S. aurata*. A dietary level of fish oil at 80 g/kg was kept constant among the diets to satisfy the n-3 essential fatty acid requirements for the species, while soybean oil was used to adjust the isoenergetic content of the diets. Corn gluten meal was used as plant source of methionine, sunflower meal was used as plant source of lysine, and wheat meal was used to adjust the isonitrogenous content of the diets. Monocalcium phosphate, vitamins E and C and a premix of vitamins and minerals were included in similar levels in all diets. Methionine and lysine were added to all insect meal-based diets to compensate deficiencies developed from fishmeal substitution.

Feedstuffs were grounded by a mill (KoMo Fidibus, PGS, Germany), and after adding oils and warm water, they were homogenised by a mixer (Maxximum MUMXL20G, Bosch) according to the dietary formulae. Pellets with 1.5 mm diameter were compounded with a California Pellet Mill (CL-2, Irmeco GmbH, Netherlands), dried at room temperature to contain moisture below 10% and preserved in air-tight bags at 4°C until used.

### 2.3. Feeding Trial

*S. aurata* juveniles of 3.4 ± 0.3 g initial mean weight were supplied by a commercial fish hatchery (Philosofish SA, Phthiotis, Greece). Juveniles were distributed randomly in triplicate glass tanks (125 L) (30 fish/tank, 3 tanks/dietary group) within a closed recirculation seawater system at the aquaculture facilities of the University of Thessaly (Laboratory of Aquaculture) and were left to acclimatise for 10-day feeding on the CTRL diet. Salt water was fixed by adding premium quality salt (Aquaforest, Poland) to reverse osmosis deionised tap water, and around 5% of water volume of each tank was changed daily by siphoning to remove wastes. During the trial, water quality parameters were monitored daily using multimeter sensors (HQ40D, Hach, USA) for temperature, pH and dissolved oxygen, an automatic temperature compenset refractometer (Blau aquaristic, Spain) for salinity and commercial test kits (API brand) for ammonia, nitrite and nitrate. Water temperature was maintained at 21.0 ± 1.0°C, dissolved oxygen >6.5 mg/L, salinity 33.0 ± 0.5 g/L, pH 8.0 ± 0.4, total ammonia nitrogen <0.2 mg/L and photoperiod 12 h light:12 h darkness. Fish were fed twice a day (09:00 and 16:00) by hand to apparent satiation for 100 days. The feed was supplied carefully with no leftovers to evaluate feed intake.

### 2.4. Sampling

Twenty fish from the initial population were taken, pooled and stored at −40°C for whole-body proximate composition analysis. Following the trial, fish underwent a 24-h starvation before sampling. The euthanisation of fish was performed with a narcotic dose (300 mg/L) of tricaine methanesulfonate (MS222) [56], and then they were individually weighed. From each tank, four fish (12 fish/dietary group) were randomly selected, their bodies were minced, and homogenates of each fish were analysed for whole-body proximate composition. Another four fish/tank (12 fish/dietary group) were sampled, and their dorsal muscle, without skin, were homogenised for muscle proximate composition. The liver and viscera from each fish were dissected and weighed for the calculation of the hepatosomatic index (HSI) and the viscerosomatic index (VSI), respectively. In addition, two fish from each tank (6 fish/dietary group) were randomly collected and their liver, and dorsal muscle tissues were stored at –80°C until their fatty acid composition was determined. For digestive enzyme analysis three fish/tank (9 fish/dietary group) were sampled after overnight fasting; their foregut, pyloric caeca and stomach including their contents were dissected out in a prechilled (<4°C) glass plate, freeze dried and stored in –80°C until analysis. For histological examination, four fish from each tank (12 fish/dietary group) were randomly sampled and dissected, and the foregut and liver tissues were fixed in Davidson fixative for 24 h and then stored in formaldehyde solution until further analysis. For intestinal microbiota analysis, the midguts of four fish per dietary group were aseptically collected after mechanically removing their digesta, and samples were stored at –80°C until analysis.

### 2.5. Growth and Feed Efficiency Indices

The equations used to calculate survival (%), weight gain (WG, g/fish), specific growth rate (SGR, %/day), feed conversion ratio (FCR), protein efficiency ratio (PER), nutrient retention (%), HSI (%), VSI (%) and condition factor (CF) are those previously described by Karapanagiotidis et al. [40]. Also, the following formulae were applied: voluntary feed intake (VFI, g/fish) = total amount of feed consumed (g) per fish; feeding rate (FR, % BW/day) = 100 × VFI (g/fish)/[(IBW + FBW)/2 × days]; and lipid retention ratio (LER) = WG (g)/lipid intake (g), where IBW and FBW are the mean initial and final body weights, respectively.

### 2.6. Proximate Composition and Amino Acid Analysis

The proximate composition of feed ingredients, including those of insect meals (FF and DF), diets, fish whole bodies and muscle tissues were performed according to the AOAC [57]. Moisture content was measured by heating in an oven at 105°C to constant weight. Crude protein content was determined by Kjeldahl analysis (behr Labor-Technik, Germany) using the value 4.76 [58] as nitrogen-to-protein conversion factor for the insect meals and 6.25 for all the other samples. Crude lipid was extracted by petroleum ether using a Soxhlet apparatus (Sox-416 Macro, Gerhard, Germany). Ash content was measured by incineration at 600°C for 5 h in muffle furnace (Nabertherm L9/12/C6, Lilienthal, Germany). Gross energy content was analysed by adiabatic calorimetry (C5000, IKA Werke, Staufen, Germany). The amino acid composition of feedstuffs and diets, except tryptophan, were performed by VELTIA (Thessaloniki, Greece, www.veltialabs.gr) using certified methods (ISO 13903:2005).

### 2.7. Fatty Acid Profiles

The fatty acid profiles of the diets, muscle and liver tissues of fish were determined as described in Karapanagiotidis et al. [59]. Briefly, the total lipid was extracted according to Folch method using chloroform:methanol (2:1, v/v). Acid catalysed transesterification of the total lipid was performed using sulphuric acid in methanol reagent (1:99, v/v). Fatty acid methyl esters (FAMEs) were purified by thin layer chromatography using glass plates precoated with silica gel and stored in isohexane containing 0.01% BHT under nitrogen at –80°C. FAMEs were separated in a capillary column (30 m × 0.25 mm id, film thickness 0.25 μm, Perkin Elmer, Waltham, MA, USA) by gas chromatography (Perkin Elmer Clarus 680, Waltham, MA, USA) using hydrogen as a carrier gas and a flame ionisation detector. Chromatograms were analysed using TotalChrom software (v. 6.3, Perkin Elmer), and identification of individual FAME was performed by comparison to known standards (FAME MIX 37, Sigma-Aldrich, St. Louis, Missouri, USA).

The following indices were determined to assess the lipid nutritional quality of the muscle tissue as proposed by Ulbricht and Southgate [60] and Santos-Silva et al. [61]: index of atherogenicity (IA) = (12:0 + 4 × 14:0 + 16:0) / (MUFA + n-3 PUFA + n-6 PUFA); index of thrombogenicity (IT) = (14:0 + 16:0 + 18:0) / [(0.5 × MUFA) + (0.5 × n-6 PUFA) + (3 × n-3 PUFA) + (n-3 PUFA/n-6 PUFA)]; and hypocholesterolemic/hypercholesterolemic (H/H) ratio = (18:1n-9 + PUFA) / (12:0 + 14:0 + 16:0).

### 2.8. Digestive Enzyme Activities

The enzyme activities of bile salt-activated lipase, total alkaline protease, trypsin and α-amylase were measured in the foregut, stomach and pyloric caeca tissues, whereas that of pepsin was measured in the stomach. The analysis was performed at IRTA (Spain) as described in Solovyev and Gisbert [62] and Tampou et al. [63], and the method description partly reproduces their wording. Briefly, for each tissue, pooled samples per dietary group were homogenised in distilled water (4°C) using an Ultra-Turrax tissue disrupter (T 25 digital, IKA-Werke, Staufen, Germany), centrifuged (3300 × *g*, 3 min at 4°C), and the supernatant was aliquoted and frozen at –80°C. Trypsin was measured using BAPNA as a substrate in 50 mM Tris-HCl, 20 mM CaCl_2_ buffer (pH 8.2). Azo-casein substrate (0.5%, w/v) in 50 mM Tris-HCl buffer (pH 8.0) was used to measure total alkaline proteases. Soluble starch (0.3%) dissolved in Na_2_HPO_4_ buffer (pH 7.4) was used a substrate to determine α-amylase. Bile salt-activated lipase activity was determined using p-nitrophenyl myristate as substrate in 0.25 mM Tris-HCl (pH 7.9), 0.25 mM 2-methoxyethanol and 5 mM sodium cholate buffer. The activity of all enzymes was determined at 25°C in duplicate per sample using a spectrophotometer (Tecan Infinite M200, Männedorf, Switzerland).

### 2.9. Histological Examination

Liver and foregut samples were subjected to serial dehydration in ethanol and immersed in xylol using a Histokinette (Leica Biosystems TP 1020, USA) and then embedded in paraffin using a modular tissue embedding system (Leica Biosystems EG1150 C, USA). Sections were cut at 4–7 μm using a microtome (SLEE medical GmbH, Mainz, Germany), deparaffinised, stained with haematoxylin and eosin and examined under light microscopy (Bresser Science TRM 301, Rhede, Germany) using a digital camera (Bresser MikroCam 5.0 MP, Rhede, Germany) under magnification of 100× and 400×. Histological alterations such as cell structure, nuclear displacement, lipid vacuolisation, haemorrhages and steatosis were assessed using a five-grade severity score [49]: 0 (not remarkable), 1 (minimal), 2 (mild), 3 (moderate) and 4 (severe). The length and width of the intestinal folds, the goblet cell number per intestinal fold and the muscular layer width of the foregut were measured from the microscope images. Five measurements per fish, and in total 60 measurements per dietary group, were performed.

### 2.10. Intestinal Microbiota Analysis

The midgut bacterial diversity was evaluated by high-throughput sequencing of the V3–V4 16S rRNA gene region as described in Meziti et al. [64]. Bulk DNA was extracted using QIAamp DNA Mini kit (QIAGEN, Germany). The 16S rRNA was PCR amplified with the primer pair S-D-Bact-0341-b-S-17 (5′-CCTACGGGNGGCWGCAG-3′) and S-D-Bact-0785-a-A-21 (5′-GACTACHVGGGTATCTAATCC-3′) on an Illumina MiSeq sequencer (2 × 300 bp) by MRDNA (Texas, USA, http://www.mrdnalab.com/). Raw sequence data were analysed with the MOTHUR software (v.1.48.0) [65]. Raw data are available from the Sequence Read Archive (https://www.ncbi.nlm.nih.gov/sra/) in BioProject PRJNA955917. Data for the FF-49 group are not presented as the samples were unfortunately damaged.

### 2.11. Statistical Analysis

The statistical analysis of microbiota analysis was performed with the PAST software (v.4.16), while for all other data the SPSS statistical software (IBM SPSS Statistics, v.29.0.0.0) was used. The normality of the data was tested by Shapiro–Wilk's test and the homogeneity of variance by Levene's test, being transformed whenever required. Means were subjected to one-way ANOVA, and Tukey's post hoc test was used for multiple comparisons. Differences were considered as significant at *p* < 0.05.

## 3. Results

### 3.1. Fish Growth and Feed Utilisation

Survival, growth performance, feed utilisation and morphometric parameters of *S. aurata* fed the experimental diets are shown in Table 4. Survival was high (91.1%–97.7%) and similar (*p* > 0.05) among the fish groups. All groups of fish had also similar VFI (g/fish) and FR (% BW/day). Moreover, final body weight, WG, SGR, FCR, PER, HSI, VSI and CF were not significantly different among the fish groups. There was a tendency of increasing LER values with increasing dietary levels of both FF and DF meals with fish fed the DF-174 diet showing a significantly higher LER than the CTRL. Protein retention was also similar in all groups, but a trend of higher lipid retention values was observed in fish fed the DF-based diets compared to fish fed the FF-based diets with the DF-58 group having a significantly higher value than the FF-49 fish.

### 3.2. Proximate Composition of Fish

The proximate composition of the whole body and muscle tissue, as well as the hepatic lipid content, of fish fed the experimental diets is shown in Table 5. Although all groups of fish had similar body lipid and energy contents, there were some significant differences among them in their moisture, protein and ash contents. In particular, fish fed the FF-49 diet had higher (*p* < 0.05) moisture and crude protein contents compared to the DF-58 fish, while the DF-174 fish had a higher (*p* < 0.05) ash content compared to the CTRL group. The muscle tissue composition was unaffected (*p* > 0.05) by the diets except the energy content that was higher in FF-97 fish compared to the CTRL fish. FF-49 and FF-97 fish had increased lipid contents in their livers, but these values were not significantly higher than those in the other groups.

### 3.3. Fatty Acid Composition of Fish

The muscle and liver fatty acid profiles of fish are shown in Tables 6 and 7, respectively. The increasing dietary inclusion of both FF and DF meals resulted in gradually increased levels of 16:0 and total SFAs in fish muscle with the DF-174 fish having significantly higher levels than the CTRL fish. Similarly, the levels of 18:1n-9 and total monounsaturated fatty acids (MUFAs) were gradually increased as the inclusion levels of FF and DF meals increased in the diet, whereas fish fed the FF-97 diet showing significantly higher values than the CTRL. On the other hand, all fish fed the *Z. morio*-based diets (FF and DF) had decreased levels of 18:2n-6 (linoleic acid [LA]) and total n-6 polyunsaturated fatty acids (PUFAs) compared to the CTRL group while the lowest (*p* < 0.05) values being observed in FF-97 and DF-174 fish. All fish fed the *Z. morio*-based diets had also slightly lower levels of 18:3n-3 (linolenic acid [LNA]), whereas fish fed the FF-97 diet showed significantly lower values of LNA than the CTRL group. A trend of reduced muscle levels of 20:5n-3 (eicosapentaenoic acid [EPA]), 22:6n-3 (docosahexaenoic acid [DHA]) and total n-3 PUFA with increasing dietary inclusions of FF and DF meals was observed, but this was not significant, while the ratio n-3/n-6 was similar in all groups. Regarding the fatty acid quality indices of the edible muscle tissue (Table 6), the DF-174 fish had higher (*p*  < 0.05) IT and lower (*p*  < 0.05) H/H than the CTRL group, while the IA was similar among the groups, although it tended to be higher in DF-174 fish.

As far as the hepatic fatty acid profiles of fish fed the experimental diets is concerned (Table 7), similar trends to those found in muscle were observed. All dietary groups had similar (*p* > 0.05) levels in all individual SFAs as well as in total SFA. However, all fish fed the *Z*. *morio*-based diets had increased total MUFA, due to their increased levels of 18:1n-9, that was significantly higher in FF-97 fish compared to the CTRL group. On the other hand, all fish fed the *Z. morio*-based diets had decreased (*p* < 0.05) total n-6 PUFA compared to the CTRL group, mainly due to their lower levels in LA. All fish fed the *Z. morio*-based diets had also slightly lower levels of LNA with FF-97 being significantly lower than the CTRL. A trend in reduced hepatic levels of EPA, DHA and total n-3 PUFA with increasing dietary inclusions of FF and DF meals was observed, but this was not significant, while the ratio n-3/n-6 was similar in all groups (*p*  > 0.05).

### 3.4. Digestive Enzyme Activities

All fish groups had similar (*p* > 0.05) trypsin and total alkaline protease activities in their foregut and pyloric caeca, whereas pepsin in the stomach did not differ (*p* > 0.05) among the dietary groups (Table 8). Regarding intestinal brush border enzymes, no differences (*p* > 0.05) were found in aminopeptidase and maltase activities. The graded dietary levels of FF and DF meals in the experimental diets led to increased α-amylase activities compared to the CTRL group that were significantly higher in the foregut of the DF-fed fish. Similarly, the increased dietary inclusion of insect meals led to significantly higher bile-salt activated lipase activities in both the foregut and pyloric caeca of fish with the highest values found in the FF-97 fish.

### 3.5. Liver and Foregut Histomorphology

**Figure 1:**
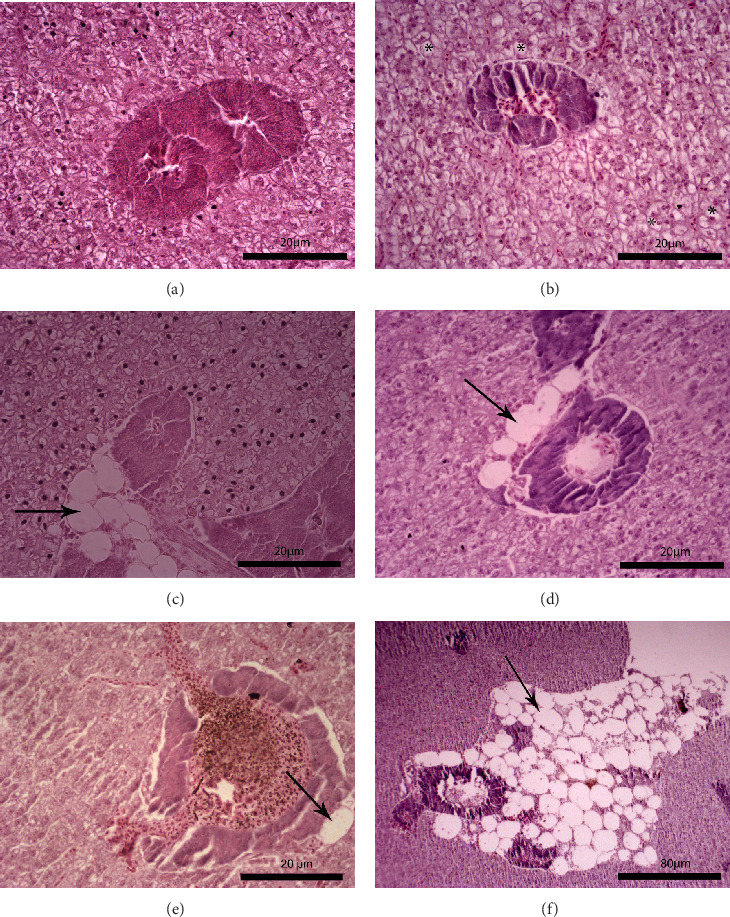
Liver histological examination. (A) Fish fed the CTRL diet: normal liver histology. (B) Fish fed the DF-58 diet: medium-sized lipid droplets (*⁣*^*∗*^) were detected in the hepatocytes in some fish. (C) Fish fed the DF-116 diet: large lipid droplets (arrow) were noticed around the pancreatic islets in some fish. (D) Fish fed the DF-174 diet: large lipid droplets (arrow) were noticed around the pancreatic islets in some fish. (E) fish fed the FF-49 diet: large lipid droplets (arrow) were accumulated around the pancreatic islets in all fish. (F) Fish fed the FF-97 diet: many large lipid droplets (arrow) were accumulated around the pancreatic islets in all fish.

**Figure 2:**
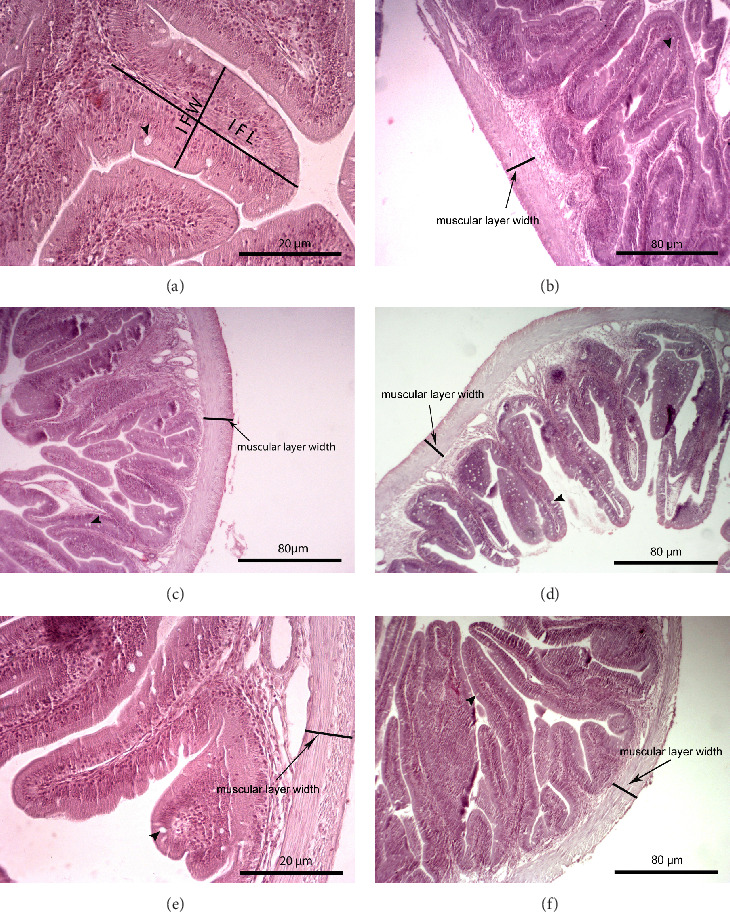
Foregut histological examination. (A) Fish fed the CTRL diet, (B) fish fed the DF-58 diet, (C) fish fed the DF-116 diet, (D) fish fed the DF-174 diet, (E) fish fed the FF-49 diet and (F) fish fed the FF-97 diet. IFL, intestinal fold length; IFW, intestinal fold width. Goblet cells are pointed with arrowhead.

The liver histology of fish fed the CTRL, DF-58, DF-116 and DF-174 diets appeared normal with minimal or mild histological changes detected (Table 9). In most of these fish, small and medium lipid droplets were observed within hepatocytes along the hepatic parenchyma, while in few cases, larger lipid droplets were noticed around the pancreatic islets (Figure 1). Moderate histological changes were detected in the liver of FF-49 and FF-97 fish (Table 9); these fish had larger lipid droplets that were accumulated around the pancreatic islets (Figure 1). The histomorphology of the foregut of all fish groups appeared normal (Figure 2). The length and width of the intestinal folds, the muscular layer width and the goblet cell number per intestinal fold of the foregut of fish are presented in Table 9. All fish groups had similar (*p* > 0.05) intestinal fold width and muscular layer width. Fish fed all the *Z. morio*-based diets had a larger length of intestinal folds with DF-174 fish being significantly different than the CTRL group. The number of goblet cells per intestinal fold was significantly reduced in fish fed the FF-49, FF-97, DF-116 and DF-174 diets compared to their congeners fed the CTRL and DF-58 diets.

### 3.6. Intestinal Microbiota

**Figure 3:**
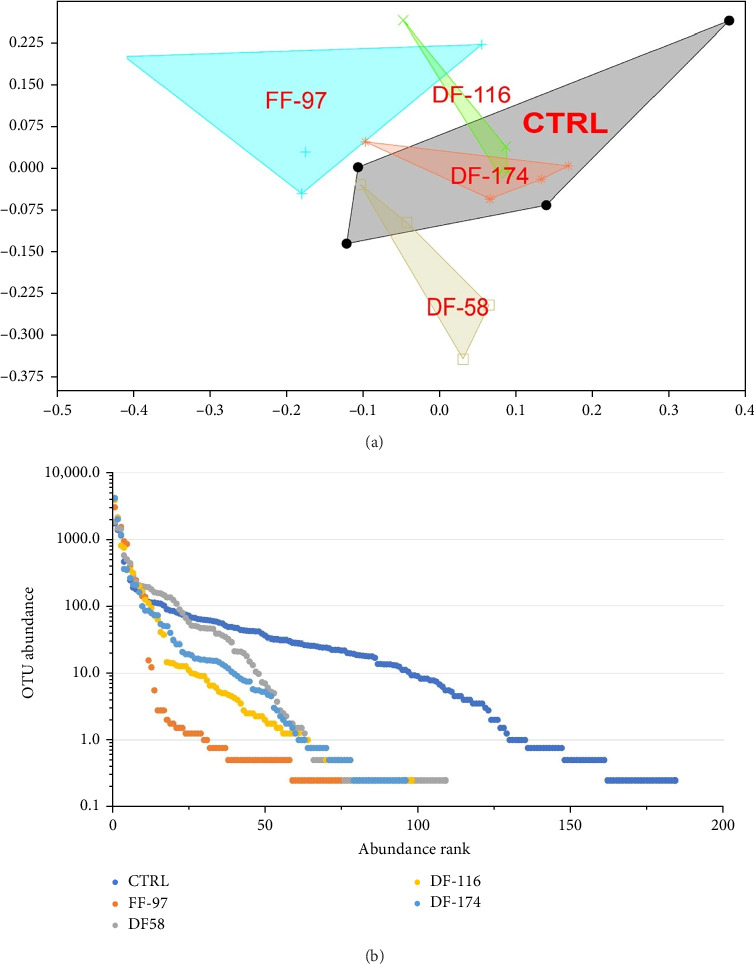
(A) Nonparametric multidimensional scaling of the gut bacterial microbiota of the dietary groups; (B) rank abundance curves of the gut bacterial operational taxonomic units (OTUs) from all dietary groups.

**Figure 4:**
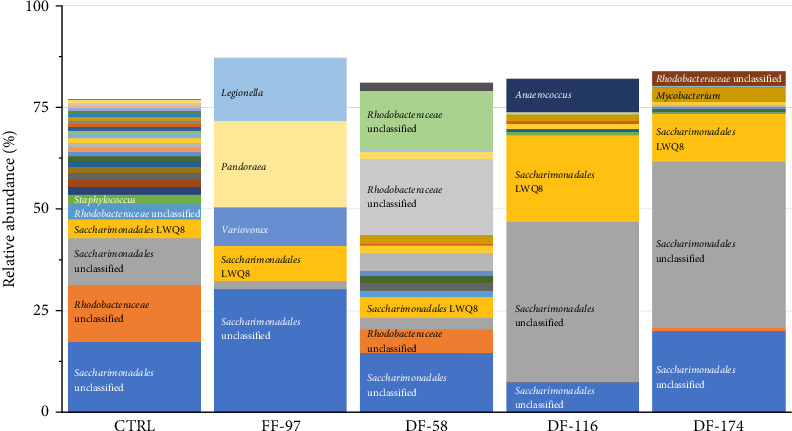
The most abundant (*Σ*_relative abundance_ ≥ 75%) gut bacterial operational taxonomic units (OTUs) in the dietary groups.

The bioinformatics analysis returned 9910 sequence reads per sample (after data normalisation) corresponding to 295 unique bacterial operational taxonomic units (OTUs). The highest number of OTUs was observed in the CTRL group (184) and the lowest (84) in FF-97. In the DF-fed groups, the number of gut bacterial OTUs ranged between 96 and 109 OTUs. The similarity of the bacterial midgut microbiota among the different dietary groups was illustrated by unconstrained ordinations using nonmetric multidimensional scaling (NMDS) (Figure 3a). The FF-97 group had a slightest overlap with the DF-116 group, while all the DF-fed groups had some overlap with the CTRL. This was also confirmed by the rank abundance curves (Figure 3b). The observed OTUs (Figure 4) were assigned to the following bacterial phyla (in descending order: *Proteobacteria* [*α*- and *γ*-*Proteobacteria*], *Actinobacteriota*, *Firmicutes*, *Bacteria* unclassified, *Bacteroidota*, *Patescibacteria*, *Acidobacteriota*, *Campylobacterota*, *Chloroflexi*, *Cyanobacteria*, *Dependentiae*, *Desulfobacterota*, *Gemmatimonadota*, *Myxococcota*, *Planctomycetota* and *Verrucomicrobiota*. The topmost abundant OTUs were yet-unaffiliated *Saccharimonadales* and *Rhodobacteraceae*. Other OTUs with relative abundance ≥10% were affiliated to the genera *Pandoraea* and *Legionella* (in FF-97 group).

## 4. Discussion

### 4.1. Fish Growth and Feed Utilisation

This research investigated the metabolic consequences of the inclusion of FF and DF *Z. morio* larvae meals replacing fishmeal in the diet of juvenile gilthead seabream. To our knowledge, this is the first study evaluating the use of *Z. morio* meal on the growth performance of gilthead seabream. The results revealed that there were no significant differences among the dietary treatments regarding final body weight, SGR, FCR, PER and other morphometrics. This, in turn, indicated that the protein quality, metabolic utilisation and growth promoting effects of *Z. morio* meals are comparable to that of fishmeal. This outcome is really promising since it signifies that juvenile seabream's growth performance and feed efficiency were not impaired by the partial replacement of fishmeal with either a dietary inclusion of FF *Z. morio* meal up to 97 g/kg replacing fishmeal protein at 10% or DF *Z. morio* meal up to 174 g/kg replacing fishmeal protein at 30%. These findings agree with relevant studies of the use of superworm meal in other fish species. For instance, dietary inclusions as high as 300 g/kg of FF *Z. morio* meals totally replacing either fishmeal [41] or soybean meal [45] did not impair the growth and feed utilisation in juvenile Nile tilapia (*O*. *niloticus*), while lower dietary levels (75–150 g/kg) resulted in even better performance [41]. In cobia (*R*. *canadum*), the dietary inclusion at 300 g/kg of a DF *Z. morio* meal, replacing 30% of fishmeal, led to similar fish growth performance and feed efficiency to the CTRL group [46]. In juvenile Asian seabass (*L*. *calcarifer*), diets using up to 120 g/kg of a DF *Z. morio* meal, replacing 44% of fishmeal, did not cause any reduction on growth and feed efficiency [51].

On the other hand, some adverse effects on zootechnical indices have been reported with the dietary use of superworm meal in other fish species. While the inclusion of a hydrolysed FF *Z. morio* meal at 100 g/kg (44% fishmeal substitution) in the diet of sea trout (*S*. *trutta m. trutta*) did not impair growth performance and FCR, it led to a significantly decreased PER value and higher HSI and VSI that the authors attributed to the chitin's presence in the insect meal [52]. Lin et al. [54] included a DF meal of *Z. atratus* up to 207.5 g/kg (up to 75% of fishmeal substitution) in the diet of Pacific white shrimp (*P*. *vannamei*), and even though no negative effects on growth performance were reported, feed efficiency was negatively impacted by all dietary inclusion levels of the insect meal (41.5–207.5 g/kg). Stathopoulou et al. [49], working with European seabass (*D*. *labrax*), reported a gradual reduction of fish growth and feed efficiency as the dietary inclusion of a DF *Z. morio* meal was increased. Hosseini Shekarabi et al. [48] reported that the growth of rainbow trout (*O*. *mykiss*) and feed efficiency were adversely affected when fish were fed on diets with 220 g/kg (40% fishmeal replacement) of a partially DF *Z. morio* meal. The former authors estimated that the optimum inclusion levels of *Z. morio* meal in the diet for rainbow trout were comprised between 162 and 184 g/kg. A decreased SGR and an increased FCR were found in perch (*P*. *fluviatilis*) when fed a diet with a mixture 1:1 of *Z. morio* and house cricket (*Acheta domesticus*) meals replacing 25% of the dietary inclusion of fishmeal, which were attributed to the lower feed consumption by fish [53].

It is generally accepted that the VFI is a key factor affecting the growth performance of fish as the higher the consumption, the higher nutrient and energy intake by fish. In gilthead seabream, for example, it has been shown that the dietary fishmeal substitution by *H. illucens* meal [40, 66–69] or *T. molitor* meal [66] significantly reduced the VFI, which in turn negatively affected growth performance and feed utilisation. However, several other studies revealed an insignificant effect [70–75]. A lower feed intake of insect-based diets has been linked to several factors not mutually exclusive like their possible lower palatability, which may be due to their organoleptics [9, 11], their high chitin content [76], their fat susceptibility to oxidation [11] and even to the processing of feed [9, 53]. In the present study, all fish groups promptly accepted the experimental diets with both forms of the insect meal not affecting the VFI and FR. This finding highlights that both FF and DF *Z. morio* meals, at the dietary inclusion levels tested here, had a similar acceptability and palatability to fishmeal for juvenile *S. aurata*. It also denotes that the defattening of *Z. morio* meal did not enhance the feed acceptability by seabream as it has been observed with *H. illucens* meal in this fish species [40] and with *Musca domestica* meal in catfish (*Clarias gariepinus*) [77]. An unaffected feed intake with the use of dietary *Z. morio* meal has also been observed in Nile tilapia [45], European seabass [49], Asian seabass [51] and sea trout [52]. On the other hand, a reduced feed intake was found in rainbow trout [48] and perch [53] using *Z. morio* meals. However, these differences between studies and species might not be exclusively related to the substitution of fishmeal by this insect meal, and other factors like the composition of experimental feeds in terms of palatable ingredients also need to be considered, which impairs the direct comparison between studies.

### 4.2. Proximate Composition and Fatty Acid Profiles

There were some alterations in the body and muscle tissue proximate compositions among the fish groups but without indicating a clear correlation with the form and inclusion level of the *Z. morio* meal in the diet. It should be noted that apart from the tested feedstuff and fish species of concern, the nutrient deposition in fish body is affected by various factors including dietary nutrient composition, feed intake and nutrient digestibility amongst others [78, 79], while the percentage of each nutrient is inversely related with the others [80]. For instance, Jabir et al. [41] incorporating a FF *Z. morio* meal up to 300 g/kg totally replacing fishmeal in the diet of Nile tilapia reported an unaffected body proximate composition. However, Alves et al. [45] found in the same species that dietary inclusion of a FF *Z. morio* meal up to 300 g/kg replacing soybean meal led to higher body moisture and lipid and lower protein and ash contents. Besides, the inclusion of a DF *Z. morio* meal up to 220 g/kg replacing fishmeal in the diet of rainbow trout significantly reduced the body protein, ash and moisture and increased the body lipid contents of rainbow trout [48].

As far as the effects of the experimental diets on the fatty acid profiles of juvenile gilthead seabream are concerned, the increasing dietary inclusions of both FF and DF *Z. morio* meals tended to increase the levels of 16:0 and 18:1n-9 and to decrease the levels of LA and LNA in both the muscle and liver tissues of fish. It should be stated, however, that this effect was only significant at the highest dietary inclusions of *Z. morio* meals and especially for the FF meal. As fishmeal contains less 16:0 than the superworm meals (Table 1), its substitution led to higher levels of this SFA in the diet (Table 3) that in turn influenced the tissue levels of fish. *Z. morio*, as most insects, is known for being rich in 18:1n-9 that can account for up to 300 mg/kg [11, 33, 52, 53]. Thus, the increased tissue levels of 18:1n-9 were due to the higher levels of this fatty acid in *Z. morio* meal, especially in the FF form, and the corresponding diets. Like vertebrates, insects can endogenously synthesise the common C16 and C18 SFA and MUFA from nonlipid precursors, but most species are unable to synthesise LA and LNA, thus displaying a dietary requirement for PUFA [81]. Although this has not yet been proved for *Z. morio*, it is most likely that the body LA and LNA of the superworm originated from their grain-based diet. Oonincx and Finke [34] pointed out that farmed insects contain high levels of LA but much lower levels of LNA than their wild counterparts due to their feeding on grains that are deficient in the latter fatty acid. This was also true for the *Z. morio* meals used in the present study, which contained much higher levels of LA, but similarly low levels of LNA as found in fishmeal (Table 1). It would be expected that the dietary incorporation of *Z. morio* will increase the tissue LA levels, but the opposite trend was observed. This is attributed to the decreasing dietary inclusion of soybean oil in the *Z. morio*-based diets, in the context of testing iso-energetic diets, since this plant oil contains about 51% of LA [82], and thus substantially influenced the fatty acid profiles of the diets and fish tissues.

The majority of terrestrial insects, including *Z. morio*, contain only traces, if any, of EPA and DHA [9, 11, 35] unless they are grown on n-3 PUFA-enriched diets [33, 83]. This was also true for the *Z. morio* meals used here, as EPA and DHA were found in traces in the FF meal and were not detected in the DF one (Table 1). This lack of EPA and DHA in terrestrial insect fat together with their high content of SFA confers a disadvantage for their use in aquafeeds, and thus, the defattening of insect meals is usually considered preferable [40]. In the present study, the lack/traces of EPA and DHA in both forms of *Z. morio* meal lowered the levels of these fatty acids in the corresponding diets (Table 2) and likewise tended to decrease their contents in fish tissues (Tables 6 and 7), but this was insignificant. It seems that the constant levels of fish oil among the diets maintained the tissue levels of EPA and DHA without compromising fish performance and condition. This suggests that dietary formulation strategies for fishmeal substitution by insect meals should contain an adequate level of fish oil in order to both satisfy the fatty acid requirements and the lipid nutritional value of fish. Similarly to our findings, Khalili Tilami et al. [53] found that the EPA and DHA levels in the fillet of perch were not significantly reduced when fish were fed on a fish oil-based diet with a mixture of superworm and house cricket meals replacing fishmeal, but their dietary strategy led to increased LA and n-6 PUFA in fish tissues. Turek et al. [47] observed that the feeding of rainbow trout on live *Z. morio* larvae, partially replacing the dietary energy of commercial pellets, was associated with lower contents of MUFA and n-3 PUFA (LNA and EPA) and higher levels of SFA in the form of 18:0 in fish fillets. The aforementioned changes in the fatty acid profiles of *S. aurata* were also reflected in the indices of IA and IT and the H/H ratio. These commonly used fatty acid indices assess the health-promoting nutritional value of food products [84] with lower IA and IT values and higher H/H ratios to be considered favourable. In the present study, the graded dietary levels of both *Z. morio* meals tended to increase the IA and IT values and decrease the H/H ratios in fish. Although these values are commonly found in fish [84], this finding indicates that the use of *Z. morio* meals deteriorated the lipid quality of fish for the consumer.

### 4.3. Digestive Enzymes

As experimental diets were isonitrogenous, the similar activities of pancreatic, gastric and intestinal proteolytic enzymes (i.e. trypsin, pepsin, total alkaline proteases and aminopeptidase) found in all fish groups denote a similar protein digestibility among the experimental diets. This in turn suggests that the protein digestibilities of both FF and DF *Z. morio* meals are similar to that of fishmeal for *S. aurata*, which explains the similar growth, protein efficiency and protein retention among the different groups. It has been shown that the protein apparent digestibility of the diet was not affected by the inclusion of superworm meal at 195 g/kg in gilthead seabream [50], at 300 g/kg in cobia [46] and up to 207 g/kg in Pacific white shrimp [54], while it was increased in European seabass at 195 g/kg [50]. On the other hand, the protein apparent digestibility was gradually reduced in response to incremental dietary levels of *Z. morio* meal in red tilapia [42], rainbow trout [48] and Asian seabass [51]. Hosseini Shekarabi et al. [48] found that the gradual dietary increase of a DF *Z. morio* meal did not affect the pepsin and chymotrypsin activities in the intestine of rainbow trout, but the trypsin activity was significantly decreased at a dietary inclusion of 220 g/kg. Lin et al. [54] reported an unaffected trypsin activity in the hepatopancreas of Pacific white shrimp, but the intestinal trypsin activity was significantly decreased at high fishmeal substitution levels by *Z. atratus* meal.

In contrast to the proteolytic activities, the increasing dietary inclusions of both FF and DF *Z. morio* meals led to a gradual increase in the bile salt-activated lipase activity in both the foregut and pyloric caeca of juvenile gilthead seabream, with the higher values found in fish fed the highest level of the FF *Z. morio* meal (FF-97). This result indicates a possibly lower lipid digestibility of *Z. morio* meal and that higher lipase activity was required to hydrolyse the insect fat fraction of the feed. It seems, however, that this effort was efficient as fish growth performance and feed conversion were not impaired by the dietary inclusion of *Z. morio* meals. This is also supported by the fact that the increasing dietary inclusions of *Z. morio* meals led to concomitant increases in lipid efficiency ratios (LERs), which showed that the ingested lipid was efficiently utilised for growth even when high amounts of *Z. morio* meal were incorporated in the diet. Lin et al. [54] also found higher lipase activity in the hepatopancreas, but not in the intestine, of Pacific white shrimp when fed a DF superworm (*Z. atratus*) meal replacing dietary fishmeal. A similar lipid digestibility of *Z. morio* meal with that of fishmeal has been shown for gilthead seabream, European seabass and Asian seabass [50, 51], but studies in red tilapia [42] and rainbow trout [48] showed that fishmeal substitution by this insect meal can significantly reduce the lipid digestibility of the diet.

Furthermore, the increasing dietary inclusions of both FF and DF *Z. morio* meals led to a gradual increase in the α-amylase activity in both the foregut and pyloric caeca of *S. aurata*, with the highest values found in fish fed the highest level of the DF *Z. morio* meal (DF-174). This result indicates a greater effort by these fish to hydrolyse the higher dietary starch into smaller polysaccharide chains. On the other hand, maltase activity was not different among the groups denoting a similar effort for disaccharide hydrolysis for energy supply. Indeed, the carbohydrate contents of *Z. morio* meals are higher than that of fishmeal (Table 1), while *Z. morio*-based diets had higher carbohydrate contents than the CTRL (Table 2) also due to the increased inclusion of wheat that was used to counterbalance isonitrogenous and isoenergetic diets. However, the carbohydrate digestibility of *Z. morio*-based diets did not exert a negative influence on fish growth and feed efficiency. Opposite to our results, the fishmeal substitution by superworm meal did not influence the α-amylase activity in Pacific white shrimp [54]. Furthermore, Mastoraki et al. [50] evaluating five different insect meals (*Z. morio*, *T. molitor*, *H. illucens*, *M. domestica* and *Alphitobius diaperinus*) found that *Z. morio* meal was the only one with a similar dietary fibre digestibility to that of fishmeal for gilthead seabream and European seabass. It is often suggested that the nutrient digestibility of insect-based diets can be reduced by the presence of chitin [5], a nitrogen-containing polysaccharide, and some authors recommend a chitinase treatment to improve the nutritional value of insect meals for their use in aquafeeds [85]. The chitin content of *Z. morio* larvae is usually at 3.9%–6% of dry matter [30], but it can be even higher. For instance, Fontes et al. [44] reported a much higher chitin content (22.5% of dry matter) in *Z. morio* compared to *T. molitor* (12.0% of dry matter) and that the *Z. morio*-based diet had a lower protein and chitin digestibilities than those of the *T. molitor*-based diet in Nile tilapia.

### 4.4. Liver and Foregut Histomorphology

The examination of possible histomorphological alterations of liver and intestine is helpful for assessing the suitability of an alternative dietary protein, such as *Z. morio* meal, as the first is the main nutrient storage organ and the latter the principal site of nutrient digestion and absorption. The present study revealed that the dietary use of *Z. morio* meals led to some mild to moderate histomorphological changes in the target tissues of juvenile *S. aurata* that were more pronounced in fish fed the FF form at the highest inclusion level used. The larger lipid droplets in the liver of these fish are consistent with their increased hepatic lipids and the lower digestibility of the fat fraction of *Z. morio* meal, as discussed previously. Moreover, their longer intestinal folds indicate an effort to increase the surface area for nutrient absorption due to a possible lower feed digestibility. Furthermore, fish fed the *Z. morio*-based diets had lower numbers of goblet cells in their foreguts, which might denote a lower mucus secretion that in turn indicates a lower promotion of the digestion process and a higher risk for intestinal inflammation. However, under the applied experimental conditions, the liver and foregut of examined fish looked mostly normal with no remarkable histopathological alternations to indicate a potentially negative effect of FF and DF *Z. morio* meals on the digestive organs. Our knowledge on the effects of dietary superworm meal on the histomorphology of fish is extremely limited. From the very few studies that have been conducted up to date, it has been reported that the foregut histomorphology (villus length-width-area, muscular layer thickness) of sea trout was not affected when fishmeal was replaced by 100 g/kg of a hydrolysed *Z. morio* meal [52]. On the other hand, a high fishmeal substitution by 166 g/kg of *Z. atratus* meal led to atrophied hepatopancreas with irregular lumen deformation in Pacific white shrimp [54].

### 4.5. Midgut Microbiota

The interplay between diet and microbiota in fish is an intricate and dynamic interaction pivotal for the overall health and welfare. The dietary choices of fish exert a major influence on the composition and activity of their gut microbiota, with wide-ranging implications for host physiology, metabolism, immune function and overall performance. Any disturbance in microbial composition or disruptions in host–microbe interactions, referred to as dysbiosis, can lead to digestive and systemic imbalances, potentially resulting in poor health and increases disease risk [86, 87]. The present study revealed that the found OTUs belonged to bacterial phyla commonly found in farmed fish [88]. The gut bacterial community of fish fed on the FF *Z. morio* meal was very different compared to the other dietary groups as it contained less than half of the total OTUs number compared to the CTRL group and high relative abundances of the *Pandoraea* and *Legionella* genera. The high abundance of *Pandoraea* sp. is surprising as these bacteria are rarely reported in fish and are known to degrade aromatic compounds such as lignin [89]. This genus cannot be linked to the fibre/chitin content of *Z*. *morio* as the DF meal had higher levels than FF meal, and thus, the DF-fed fish should have also showed increased abundances of *Pandoraea* sp., but that was not the case. This genus was dominant during the early development stage of Yangtze sturgeon, *Acipenser dabryanus* [90], and it has been considered as potentially beneficial due to its decreased relative abundance after a parasitic infection in orange-spotted grouper (*Epinephelus coioides*) [91]. *Pandoraea* genus has also been linked with the modulation of lipid metabolism in zebrafish (*Danio rerio*) after feeding on ginseng fermentation alcoholic solution [92]. The latter role may explain the higher abundance of these bacteria in fish fed the FF *Z. morio* meal in opposition to the other groups.

Members of the *Legionella* genera are considered as pathogenic in humans [93] and have impaired the intestinal integrity in zebrafish [94]. However, *Legionella* genera is frequently detected in aquatic environment and fish [94–96], and thus, it is likely that they play positive roles in the dynamics of fish gut microbiota [95, 97]. In our study, the fact that *Legionella* sp. was not found in the CTRL and in the DF *Z. morio*-fed groups indicates that it was either outcompeted by other bacterial species or it is related to the *Z. morio* fat fraction. An increased abundance of *Legionella* sp. has been tightly correlated to fatty acid synthesis and lipogenesis in zebrafish [98], while a decreased abundance occurred in the midgut of yellowtail (*Seriola dumerili*) when dietary fishmeal and fish oil were replaced by alternative meals and oils [96].

The most important OTUs in this study were affiliated to higher taxa only, namely, the *Saccharimonadales* and the *Rhodobacteraceae*. There is very limited information on the *Saccharimonadales* in fish gut habitats. *Saccharimonas*-related OTUs were significantly increased in rainbow trout gut microbiota feeding on polysaccharide-rich herbal feedstuffs [99, 100] and were outcompeted in the gut of juvenile olive flounder (*Paralichthys olivaceus*) when infected by three pathogenic bacteria [101]. It seems that such OTUs are not related to detrimental effects on the gut microbiome and as such they could be considered either commensals or beneficial. The fact that the OTUs found in this study were related to the *Rhodobacteraceae* family only, and were not securely attributed to a lower taxon, shows the untapped diversity of this large and diverse family of widespread bacteria in the marine environment [102]. However, the known *Rhodobacteraceae* taxa related to fish are considered beneficial [103], and thus, we suggest that the increased abundance of these OTUs in our study offers an advantageous gut microbiota profile.

Studies on the effects of *Z. morio* meal upon fish gut microbiota are extremely scarce. Mikołajczak et al. [52] reported that the fishmeal substitution by *Z. morio* meal in sea trout diet decreased the concentrations of *Carnobacterium* spp., thus indicating a higher possibility for pathogen proliferation. On the other hand, the authors reported decreased concentrations of *Aeromonas* spp., which include pathogenic and opportunistic bacteria, and *Enterococcus* spp. that are considered virulent even though they could also act as probiotics. Lin et al. [54] reported that fishmeal substitution by DF *Z. atratus* meal in the diet of Pacific white shrimp increased the abundances of *Bacteroidetes* and *Firmicutes* and decreased that of *Proteobacteria*. The authors stated that this insect meal can improve the antibacterial activity of the immune system by regulating the intestinal flora structure and its metabolic functions, but the excessive fishmeal substitution levels may negatively influence shrimp's health as the pathogenic *Flexispira* spp. abundance was increased. Certainly, much more is needed to know about the effects of dietary insect meals on the intestinal microbiota of fish and what are their implications for fish nutrition and health.

## 5. Conclusions

In conclusion, the partial substitution of fishmeal by the dietary inclusion up to 97 g/kg of FF or up to 174 g/kg of DF *Z. morio* larvae meal did not compromise the survival, growth performance and feed utilisation of juvenile gilthead seabream. The dietary inclusion of *Z. morio* meals did not significantly reduce the levels of EPA and DHA in fish tissues, but since its fat fraction is lacking these valuable fatty acids, an adequate dietary inclusion of fish oil is required to support fish growth and nutritional quality. The increasing inclusions of both insect meal forms led to gradual increases in the bile salt-activated lipase and α-amylase activities indicating a compensatory mechanism for lipid and carbohydrate digestion. Increased levels of the FF *Z. morio* meal led to mild histomorphological changes in fish with larger lipid droplets in hepatocytes, longer intestinal folds and less goblet cells. Also, the high dietary inclusion of the FF *Z. morio* meal differentiated the gut microbiota of *S*. *aurata* with high relative abundances of the *Pandoraea* and *Legionella* genera, but the DF meal induced similar midgut bacterial communities as those found in fish fed the CTRL diet being dominated by the potentially beneficial members of the *Saccharimonadales* and *Rhodobacteraceae*. No dysbiosis was observed by the partial inclusion of both *Z. morio* meals. These results indicated that *Z. morio* meal is a suitable insect protein for fishmeal substitution towards more sustainable aquafeeds for *S. aurata* that deserves more attention by researchers and insect producers.

## Figures and Tables

**Table 1:** Proximate composition (% as fed), amino acid composition (g/100 g meal) and selected fatty acids (% of total) of the tested ingredients: fishmeal (FM), full-fat (FF) *Zophobas morio* and defatted (DF) *Z. morio* meals.

Dietary groups	FM	FF	DF
Proximate composition (%)	—	—	—
Moisture	7.21	11.95	5.40
Crude protein (×4.76^a^)	—	31.26	53.00
Crude protein (×6.25)	65.50	41.04	69.40
Crude lipid	10.11	39.73	3.79
Total carbohydrates^b^	1.27	13.95	32.33
Ash	15.91	3.11	5.48
Gross energy (KJ/g)	19.17	27.19	21.71
Amino acids (g/100 g meal)			
* Essential amino acids*	—	—	—
Arginine	3.84	0.97	1.67
Histidine	1.90	1.27	2.46
Isoleucine	2.76	2.18	3.69
Leucine	4.78	2.73	4.26
Lysine	5.28	3.23	4.67
Methionine	1.74	0.28	0.40
Phenylalanine	2.68	1.59	2.49
Threonine	2.93	1.29	2.14
Valine	3.25	2.50	4.70
*Nonessential amino acids*	—	—	—
Alanine	4.09	2.26	4.24
Aspartic acid	6.15	3.86	5.66
Cysteine	0.55	0.21	0.30
Glutamic acid	8.53	2.75	5.22
Glycine	3.96	1.20	2.10
Proline	2.66	1.50	2.63
Serine	2.61	1.15	1.93
Tyrosine	2.06	2.06	3.95
Selected fatty acids (% of total)			
16:0	20.0	28.0	38.6
18:1n-9	8.2	35.9	30.9
18:2n-6	1.1	24.5	19.3
18:3n-3	0.6	1.0	0.6
20:4n-6	1.5	n.d.	n.d.
20:5n-3	17.8	0.30	n.d.
20:6n-3	8.2	0.29	n.d.
SFA^c^	35.5	34.9	46.3
MUFA^d^	29.3	38.7	33.6
PUFA^e^	35.2	26.4	20.0

Abbreviations: MUFA, monounsaturated fatty acid; n.d., not detected; PUFA, polyunsaturated fatty acid; SFA, saturated fatty acid.

^a^Nitrogen-to-protein conversion factor [58].

^b^Total carbohydrates (including fibre) = 100 − (moisture + protein + lipid + ash).

^c^Total SFAs include 14:0, 15:0, 16:0, 17:0 18:0, 20:0, 22:0 and 24:0.

^d^Total MUFAs include 16:1n-7, 18:1n-9, 18:1n-7, 20:1n-9, 22:1n-9 and 24:1n-9.

^e^Total PUFAs include 18:3n-6, 20:2n-6, 20:3n-6, 22:5n-6, 18:4n-3, 20:3n-3, 20:4n-3 and 22:5n-3.

**Table 2:** Dietary formulation (g/kg) and proximate composition (% as fed) of the control (CTRL) diet and diets where FM was replaced by full-fat (FF) or defatted (DF) *Zophobas morio* larvae meals.

Dietary groups	CTRL	FF-49	FF-97	DF-58	DF-116	DF-174
Formulation (g/kg)	—	—	—	—	—	—
Fishmeal^a^	613	582	550	553	492	431
* Zophobas* meal, full-fat	0	49	97	0	0	0
* Zophobas* meal, defatted	0	0	0	58	116	174
Wheat meal^b^	78	80	87.3	87.3	93	104
Corn gluten meal^c^	127	123.4	119	120	114	108
Sunflower meal^d^	63	62	62	62	62.7	63
Fish oil^e^	80	80	70	80	80	80
Soybean oil^f^	30	12	0	25	22	15
Vitamins and minerals, premix^g^	3	3	3	3	3	3
Monocalcium phosphate^h^	3	3	3	3	3	3
Vitamin E^i^	2	2	2	2	2	2
Vitamin C^i^	1	1	1	1	1	1
Lysine^j^	0	1.1	2.4	2.5	4.9	7.0
Methionine^j^	0	1.5	3.3	3.2	6.4	9.0
Proximate composition (%)	—	—	—	—	—	—
Moisture	8.50	8.70	8.30	8.40	8.30	8.80
Total nitrogen	8.39	8.38	8.42	8.41	8.45	8.43
Crude lipid	15.62	15.58	14.65	14.35	14.07	13.26
Total carbohydrates	11.80	12.70	13.50	13.93	15.03	16.20
Ash	11.65	10.63	10.90	10.77	9.79	9.06
Gross energy (Mj/kg)	21.14	20.99	21.38	20.99	20.98	21.12

^a^Sardine and mackerel fishmeal, Köster Marine Proteins GmbH, Hamburg, Germany.

^b^Local market.

^c^Glutalys, Roquette Italia S.p.A, Cassano Spinola, Italy.

^d^Pavlos N. Pettas S.A., Patra, Greece.

^e^Trimmings fish oil (8.6% 20:5n-3, 8.8% 22:6n-3), Distral S.A., Aspropirgos, Greece.

^f^Non-GM, Soya Hellas, Athens, Greece.

^g^Vitamin and mineral supplement (per kg of mixture): vitamins: E, 58.3 g; K3, 3.3 g; A, 1500 IU/g; D3, 200 IU/g; B1, 3.3 g; B2, 6.6 g; B6, 3.3 mg; B12, 10 mg; folic acid, 3.3 g; biotin, 100 mg; inositol, 40 g; C, 33.3 g; nicotinic acid, 16.6 g; pantothenic acid, 13.3 g. Minerals: Co, 170 mg; I, 248 mg (Ca(IO_3_)_2_); Mn, 10 g (MnO); Zn, 33 g (ZnO); Ca, 235 g; Se, 2.5 mg (Na_2_SeO_3_); Na, 247.5 mg (Na_2_SeO_3_); Fe, 2 g; Mg, 121.3; Cu, 0.8 g.

^h^Astron Chemicals SA, Attica, Greece.

^i^DSM Nutritional Products Hellas Ltd, Athens, Greece.

^j^MetAMINO (DL-Methionine Feed Grade), Evonik Nutrition & Care GmbH, Athens, Greece.

**Table 3:** Amino acid composition (g/100 g of diet) and selected fatty acids (% of total) of the experimental diets.

Dietary groups	CTRL	FF-49	FF-97	DF-58	DF-116	DF-174
Essential amino acids (g/100 g diet)						
Arginine	3.53	3.54	3.49	3.31	3.18	3.01
Histidine	1.30	1.35	1.40	1.30	1.32	1.23
Isoleucine	2.11	2.17	2.27	2.30	2.11	2.03
Leucine	4.52	4.54	4.52	4.54	4.30	4.08
Lysine	3.43	3.53	3.80	3.60	3.60	3.39
Methionine	1.48	1.61	1.69	1.58	1.72	1.64
Phenylalanine	2.27	2.38	2.43	2.30	2.20	2.10
Threonine	2.22	2.25	2.39	2.19	2.15	2.05
Valine	2.47	2.64	2.74	2.63	2.56	2.58
Nonessential amino acids (g/100 g diet)						
Alanine	3.42	3.47	3.48	3.47	3.49	3.31
Aspartic acid	4.71	4.77	4.99	4.62	4.54	4.09
Cysteine	0.53	0.55	0.53	0.50	0.50	0.53
Glutamic acid	7.82	8.03	7.97	7.73	7.57	7.38
Glycine	3.03	2.99	3.05	3.01	2.93	2.73
Proline	2.60	2.73	2.67	2.73	2.85	2.69
Serine	2.37	2.41	2.45	2.36	2.34	2.08
Tyrosine	1.75	1.96	1.94	2.01	2.19	2.08
Selected fatty acids (% of total)						
16:0	18.1	21.2	23.4	18.5	18.5	20.0
18:1n-9	23.2	25.0	26.7	24.1	24.5	25.5
18:2n-6	16.8	14.0	13.2	16.5	15.6	15.7
18:3n-3	2.9	2.3	1.8	2.6	2.4	2.6
20:4n-6	0.6	0.5	0.5	0.6	0.6	0.6
20:5n-3	7.4	6.2	5.9	7.2	6.8	6.4
22:6n-3	6.2	6.2	5.6	5.9	5.7	5.4
SFA	29.1	32.9	35.5	29.7	29.5	31.0
MUFA	36.5	37.7	38.3	37.2	38.2	38.0
Total n-6 PUFA	17.7	14.8	13.9	17.4	16.4	16.6
Total n-3 PUFA	16.6	14.6	13.3	15.7	15.9	14.4

**Table 4:** Growth performance, feed utilisation and morphometric parameters of *Sparus aurata* fed the experimental diets.

Parameters/dietary groups	CTRL	FF-49	FF-97	DF-58	DF-116	DF-174
Survival (%)	96.7 ± 3.3	97.8 ± 1.9	91.1 ± 7.7	97.8 ± 3.8	96.7 ± 3.3	95.6 ± 1.9
VFI (g/fish)	43.5 ± 1.5	44.2 ± 0.5	44.7 ± 1.3	47.7 ± 3.1	42.8 ± 1.3	45.2 ± 0.9
FR (% BW/day)	2.52 ± 0.12	2.44 ± 0.13	2.62 ± 0.16	2.52 ± 0.17	2.41 ± 0.04	2.58 ± 0.07
IBW (g/fish)	3.4 ± 0.0	3.40 ± 0.0	3.40 ± 0.0	3.40 ± 0.0	3.40 ± 0.0	3.40 ± 0.0
FBW (g/fish)	38.4 ± 2.1	40.2 ± 2.3	39.0 ± 2.2	42.2 ± 2.4	38.8 ± 1.8	39.2 ± 2.3
TL (cm)	13.1 ± 0.2^b^	13.6 ± 0.3^a,b^	13.3 ± 0.2^a,b^	13.8 ± 0.4^a^	13.3 ± 0.2^a,b^	13.4 ± 0.1^a,b^
WG (g/fish)	35.0 ± 2.1	36.8 ± 2.2	35.6 ± 2.2	38.8 ± 2.4	35.4 ± 1.8	35.8 ± 2.3
SGR (%/day)	2.42 ± 0.05	2.47 ± 0.05	2.44 ± 0.06	2.52 ± 0.06	2.44 ± 0.05	2.44 ± 0.06
FCR	1.25 ± 0.07	1.20 ± 0.08	1.26 ± 0.04	1.23 ± 0.06	1.21 ± 0.02	1.27 ± 0.06
PER	1.53 ± 0.09	1.59 ± 0.11	1.51 ± 0.05	1.55 ± 0.08	1.57 ± 0.04	1.50 ± 0.07
LER	5.15 ± 0.31^b^	5.34 ± 0.37^a,b^	5.44 ± 0.19^a,b^	5.69 ± 0.29^a,b^	5.87 ± 0.15^a,b^	5.95 ± 0.27^a^
Protein retention (%)	27.0 ± 1.8	28.0 ± 2.3	26.4 ± 1.0	27.7 ± 1.3	27.6 ± 0.9	26.3 ± 0.9
Lipid retention (%)	65.5 ± 8.5^a,b^	58.8 ± 3.3^b^	66.0 ± 5.7^a,b^	77.5 ± 2.2^a^	71.3 ± 3.5^a,b^	72.2 ± 4.0^a,b^
HSI (%)	1.58 ± 0.03	1.61 ± 0.09	1.79 ± 0.33	1.48 ± 0.13	1.61 ± 0.23	1.60 ± 0.12
VSI (%)	7.89 ± 0.17	7.74 ± 0.26	8.32 ± 1.00	8.32 ± 0.37	8.40 ± 1.29	8.03 ± 0.52
CF	1.73 ± 0.02	1.65 ± 0.10	1.63 ± 0.03	1.60 ± 0.04	1.64 ± 0.03	1.61 ± 0.07

*Note:* Values represent means ± standard deviation (*n* = 3). Values within each row not sharing a common superscript letter are significantly different (*p*  < 0.05).

Abbreviations: CF, condition factor; FBW, final body weight; FCR, feed conversion ratio; FR, feeding rate; HSI, hepatosomatic index; IBW, initial body weight; LER, lipid efficiency ratio; PER, protein efficiency ratio; SGR, specific growth rate; TL, final total length; VFI, voluntary feed intake; VSI, viscerosomatic index; WG, weight gain.

**Table 5:** Proximate composition (% of dry weight) and energy content (KJ/g) of whole body and muscle tissue and lipid content (% of wet weight) of liver tissue of *Sparus aurata* fed the experimental diets.

Dietary groups	CTRL	FF-49	FF-97	DF-58	DF-116	DF-174
Whole body composition (% DW)	—	—	—	—	—	—
Moisture (% wet weight)	67.6 ± 0.7^a,b^	69.1 ± 0.5^a^	68.0 ± 0.7^a^	65.7 ± 0.6^b^	67.9 ± 0.7^a^	67.4 ± 1.1^a,b^
Crude protein (%)	53.4 ± 1.2^a,b^	56.2 ± 1.8^a^	53.8 ± 1.1^a,b^	51.4 ± 0.8^b^	54.0 ± 2.1^a,b^	53.0 ± 1.2^a,b^
Crude lipid (%)	36.7 ± 1.9	33.8 ± 2.3	35.6 ± 1.1	37.5 ± 0.8	35.5 ± 1.2	35.0 ± 0.6
Ash (%)	9.7 ± 0.2^b^	9.9 ± 0.4^a,b^	9.9 ± 0.3^a,b^	9.9 ± 0.4^a,b^	9.7 ± 0.2^a,b^	10.6 ± 0.5^a^
Gross energy (kJ/g)	26.1 ± 0.1	25.6 ± 0.4	25.8 ± 0.2	25.9 ± 0.3	26.0 ± 0.1	25.5 ± 0.1
Muscle composition (% DW)	—	—	—	—	—	—
Moisture (% wet weight)	75.4 ± 0.3	75.0 ± 0.9	74.8 ± 0.9	74.9 ± 0.5	74.0 ± 0.5	74.9 ± 0.3
Crude protein (%)	83.3 ± 1.4	82.3 ± 1.9	81.4 ± 0.6	82.2 ± 0.9	81.3 ± 2.0	83.3 ± 1.2
Crude lipid (%)	8.40 ± 0.6	9.40 ± 1.9	10.3 ± 1.4	9.80 ± 0.7	10.7 ± 1.7	8.40 ± 0.5
Ash (%)	6.7 ± 0.3	6.6 ± 0.2	6.4 ± 0.2	6.6 ± 0.4	6.4 ± 0.2	6.7 ± 0.2
Gross energy (kJ/g)	23.1 ± 0.3^b^	23.3 ± 0.3^ab^	23.8 ± 0.2^a^	23.6 ± 0.1^ab^	23.7 ± 0.2^ab^	23.2 ± 0.1^ab^
Hepatic crude lipid (% WW)	10.8 ± 2.8	13.4 ± 3.2	14.3 ± 2.7	12.1 ± 0.8	12.6 ± 2.3	12.2 ± 2.6

*Note:* Values represent means ± standard deviation (*n* = 3). Values within each row not sharing a common superscript letter are significantly different (*p*  < 0.05).

**Table 6:** Fatty acid composition (% of total fatty acids) of the muscle tissue of *Sparus aurata* fed the experimental diets.

Fatty acid	CTRL	FF-49	FF-97	DF-58	DF-116	DF-174
14:0	2.5 ± 0.1	2.4 ± 0.2	2.1 ± 0.1	2.4 ± 0.4	2.5 ± 0.3	2.4 ± 0.2
15:0	0.3 ± 0.0	0.3 ± 0.0	0.3 ± 0.0	0.2 ± 0.0	0.3 ± 0.0	0.3 ± 0.0
16:0	17.1 ± 0.7^b^	18.6 ± 1.7^a,b^	19.1 ± 1.9^a,b^	17.5 ± 1.3^b^	18.5 ± 1.4^a,b^	20.5 ± 1.6^a^
18:0	4.1 ± 0.4	4.3 ± 0.4	4.7 ± 0.5	4.2 ± 0.2	4.2 ± 0.5	4.5 ± 0.6
20:0	0.2 ± 0.0	0.2 ± 0.1	0.2 ± 0.1	0.2 ± 0.0	0.2 ± 0.0	0.2 ± 0.0
22:0	0.1 ± 0.0	0.1 ± 0.0	0.1 ± 0.0	0.1 ± 0.0	0.1 ± 0.2	0.1 ± 0.0
SFA	24.3 ± 0.9^b^	25.9 ± 1.8^a,b^	26.4 ± 2.4^a,b^	24.8 ± 1.3^a,b^	25.8 ± 1.7^a,b^	28.0 ± 1.1^a^

16:1n-9	0.4 ± 0.1	0.6 ± 0.1	0.7 ± 0.2	0.5 ± 0.1	0.5 ± 0.0	0.5 ± 0.0
16:1n-7	4.8 ± 0.7	4.5 ± 0.6	4.6 ± 0.4	4.8 ± 0.6	4.9 ± 0.6	4.8 ± 0.4
18:1n-9	21.7 ± 1.7^b^	22.2 ± 2.9^a,b^	25.5 ± 2.5^a^	23.5 ± 1.2^a,b^	24.6 ± 1.4^a,b^	24.7 ± 0.6^a,b^
18:1n-7	3.5 ± 0.1	3.3 ± 0.3	3.3 ± 0.3	3.5 ± 0.1	3.4 ± 0.2	3.6 ± 0.0
20:1n-9	0.5 ± 0.1^a^	1.5 ± 0.2^a,b^	1.6 ± 0.2^b^	1.9 ± 0.1^b^	1.8 ± 0.1^b^	1.9 ± 0.4^b^
22:1n-9	0.4 ± 0.1	0.3 ± 0.1	0.4 ± 0.1	0.3 ± 0.0	0.4 ± 0.0	0.4 ± 0.0
24:1n-9	0.5 ± 0.1	0.5 ± 0.1	0.5 ± 0.1	0.5 ± 0.1	0.4 ± 0.1	0.5 ± 0.1
MUFA	31.7 ± 2.4	32.4 ± 4.5	36.5 ± 3.2	35.0 ± 1.9	36.0 ± 1.8	36.3 ± 0.2

18:2n-6	14.6 ± 0.8^a^	12.2 ± 1.0^b,c^	11.2 ± 1.0^c^	13.3 ± 0.7^a,b^	13.2 ± 0.4^a,b^	12.2 ± 1.2^b,c^
18:3n-6	0.3 ± 0.1	0.2 ± 0.0	0.2 ± 0.1	0.2 ± 0.1	0.2 ± 0.1	0.2 ± 0.0
20:2n-6	0.6 ± 0.1	0.5 ± 0.1	0.5 ± 0.0	0.5 ± 0.1	0.5 ± 0.1	0.5 ± 0.0
20:3n-6	0.4 ± 0.1	0.6 ± 0.1	0.5 ± 0.1	0.5 ± 0.1	0.4 ± 0.1	0.5 ± 0.1
20:4n-6	1.1 ± 0.2	1.1 ± 0.2	1.0 ± 0.1	1.0 ± 0.2	0.9 ± 0.2	0.9 ± 0.2
22:5n-6	0.3 ± 0.1	0.3 ± 0.1	0.3 ± 0.0	0.3 ± 0.1	0.3 ± 0.1	0.3 ± 0.0
n-6 PUFA	17.4 ± 0.6^a^	15.3 ± 0.8^b^	13.7 ± 1.1^c^	15.6 ± 0.7^b^	15.3 ± 0.5^b^	14.6 ± 1.3^b,c^

18:3n-3	2.3 ± 0.2^a^	1.8 ± 0.2^a,b^	1.6 ± 0.5^b^	2.2 ± 0.2^a^	2.1 ± 0.1^a,b^	1.9 ± 0.2^a,b^
18:4n-3	0.2 ± 0.0	0.2 ± 0.0	0.2 ± 0.1	0.2 ± 0.0	0.2 ± 0.0	0.2 ± 0.2
20:3n-3	0.2 ± 0.0	0.2 ± 0.0	0.2 ± 0.0	0.2 ± 0.0	0.2 ± 0.0	0.2 ± 0.0
20:4n-3	0.8 ± 0.0	0.8 ± 0.1	0.8 ± 0.1	0.9 ± 0.0	0.8 ± 0.1	0.8 ± 0.0
20:5n-3	7.4 ± 1.1	7.2 ± 1.5	7.1 ± 1.2	7.5 ± 0.8	6.8 ± 0.8	6.3 ± 0.5
22:5n-3	2.7 ± 0.4	2.6 ± 0.5	2.5 ± 0.5	2.6 ± 0.4	2.3 ± 0.3	2.2 ± 0.1
22:6n-3	11.8 ± 2.2	11.6 ± 2.9	10.4 ± 1.8	11.1 ± 2.2	9.7 ± 2.1	9.3 ± 0.8
n-3 PUFA	25.4 ± 3.5	24.4 ± 4.1	22.9 ± 3.7	24.7 ± 3.3	22.1 ± 3.1	20.8 ± 0.3

n-3/n-6	1.5 ± 0.3	1.6 ± 0.3	1.7 ± 0.3	1.6 ± 0.2	1.4 ± 0.2	1.4 ± 0.1

IA	0.36 ± 0.04	0.39 ± 0.03	0.37 ± 0.05	0.36 ± 0.04	0.39 ± 0.03	0.42 ± 0.03
IT	0.23 ± 0.02^b^	0.26 ± 0.04^a,b^	0.28 ± 0.05^a,b^	0.24 ± 0.04^a,b^	0.27 ± 0.04^a,b^	0.31 ± 0.01^a^
H/H	3.30 ± 0.23^a^	2.95 ± 0.35^a,b^	2.96 ± 0.42^a,b^	3.23 ± 0.37^a,b^	2.97 ± 0.29^a,b^	2.63 ± 0.19^b^

*Note:* Values represent means ± standard deviation (*n* = 3). Values within each row not sharing a common superscript letter are significantly different (*p*  < 0.05).

Abbreviations: H/H, hypocholesterolemic/hypercholesterolemic ratio; IA, index of atherogenicity; IT, index of thrombogenicity; MUFA, monounsaturated fatty acid; PUFA, polyunsaturated fatty acid; SFA, total saturated fatty acid.

**Table 7:** Fatty acid composition (% of total fatty acids) of the liver tissue of *Sparus aurata* fed the experimental diets.

Fatty acid	CTRL	FF-49	FF-97	DF-58	DF-116	DF-174
14:0	3.3 ± 0.2	3.0 ± 0.2	3.0 ± 0.1	3.3 ± 0.7	2.7 ± 0.8	3.3 ± 0.1
15:0	0.3 ± 0.0	0.3 ± 0.0	0.3 ± 0.0	0.2 ± 0.0	0.2 ± 0.0	0.2 ± 0.0
16:0	18.5 ± 0.7	18.5 ± 0.9	19.7 ± 0.9	19.6 ± 0.2	19.1 ± 1.1	19.4 ± 0.5
17:0	0.3 ± 0.0	0.4 ± 0.0	0.4 ± 0.0	0.3 ± 0.0	0.4 ± 0.0	0.3 ± 0.0
18:0	4.4 ± 0.4	4.5 ± 0.5	4.5 ± 0.3	4.6 ± 0.7	5.0 ± 0.7	4.9 ± 0.4
20:0	0.1 ± 0.0	0.1 ± 0.0	0.1 ± 0.1	0.1 ± 0.0	0.1 ± 0.0	0.1 ± 0.0
23:0	0.2 ± 0.0	0.1 ± 0.0	0.1 ± 0.0	0.2 ± 0.0	0.1 ± 0.0	0.2 ± 0.0
SFA	27.0 ± 0.9	27.0 ± 0.6	28.2 ± 0.8	28.3 ± 0.1	27.6 ± 1.4	27.5 ± 0.6

16:1n-7	5.8 ± 0.1	5.6 ± 0.1	5.7 ± 0.2	5.7 ± 0.4	5.3 ± 0.2	5.6 ± 0.2
18:1n-9	25.0 ± 1.0^b^	30.0 ± 1.5^a^	31.3 ± 1.9^a^	27.3 ± 1.0^a,b^	27.0 ± 2.5^a,b^	28.6 ± 1.0^a,b^
18:1n-7	4.3 ± 0.4	4.5 ± 0.6	4.5 ± 0.3	4.3 ± 0.3	4.6 ± 0.5	4.7 ± 0.2
20:1n-9	1.6 ± 0.2	1.7 ± 0.4	1.6 ± 0.2	1.4 ± 0.1	1.9 ± 0.2	2.0 ± 0.4
22:1n-9	0.7 ± 0.0	0.8 ± 0.1	0.6 ± 0.2	0.6 ± 0.0	0.7 ± 0.0	0.8 ± 0.3
24:1n-9	0.5 ± 0.0	0.5 ± 0.0	0.5 ± 0.1	0.4 ± 0.0	0.5 ± 0.0	0.5 ± 0.0
MUFA	37.9 ± 0.5^b^	43.0 ± 2.4^a,b^	44.2 ± 2.5^a^	39.8 ± 1.6^a,b^	40.0 ± 2.8^a,b^	42.2 ± 1.6^a,b^

18:2n-6	15.2 ± 0.9^a^	13.3 ± 0.3^b^	11.5 ± 0.3^c^	13.5 ± 0.9^a,b^	13.2 ± 0.7^b,c^	12.9 ± 0.4^b,c^
18:3n-6	1.0 ± 0.4	0.5 ± 0.2	1.0 ± 0.2	0.7 ± 0.3	0.8 ± 0.4	0.9 ± 0.9
20:2n-6	0.5 ± 0.0	0.5 ± 0.1	0.5 ± 0.0	0.5 ± 0.0	0.5 ± 0.0	0.5 ± 0.1
20:4n-6	0.7 ± 0.1	0.6 ± 0.0	0.6 ± 0.1	0.6 ± 0.0	0.7 ± 0.0	0.6 ± 0.0
n-6 PUFA	17.1 ± 0.2^a^	15.0 ± 0.2^b,c^	13.6 ± 0.5^c^	15.4 ± 1.1^a,b^	15.0 ± 0.8^b,c^	15.0 ± 0.6^b,c^

18:3n-3	2.0 ± 0.2^a^	1.7 ± 0.0^a,b^	1.3 ± 0.0^b^	1.9 ± 0.1^a^	1.6 ± 0.2^a,b^	1.7 ± 0.1^a^
20:3n-3	0.1 ± 0.0	0.2 ± 0.0	0.1 ± 0.0	0.2 ± 0.0	0.2 ± 0.0	0.2 ± 0.0
20:5n-3	3.7 ± 0.4	3.4 ± 0.5	3.2 ± 0.2	3.4 ± 0.5	3.5 ± 0.0	3.2 ± 0.3
22:5n-3	2.1 ± 0.2	1.9 ± 0.3	1.7 ± 0.1	2.2 ± 0.4	2.0 ± 0.3	2.0 ± 0.3
22:6n-3	10.1 ± 1.3	7.8 ± 1.8	7.6 ± 1.6	8.8 ± 1.5	9.4 ± 0.7	8.3 ± 1.3
n-3 PUFA	17.9 ± 1.0	15.0 ± 2.2	14.0 ± 1.8	16.5 ± 2.3	16.5 ± 0.8	15.4 ± 1.2

n-3/n-6	1.0 ± 0.1	1.0 ± 0.2	1.0 ± 0.1	1.1 ± 0.2	1.2 ± 0.0	1.0 ± 0.0

*Note:* Values represent means ± standard deviation (*n* = 3). Values within each row not sharing a common superscript letter are significantly different (*p*  < 0.05).

Abbreviations: MUFA, monounsaturated fatty acid; PUFA, polyunsaturated fatty acid; SFA, total saturated fatty acid.

**Table 8:** Digestive enzymes activities (mU/mg protein) and protein concentration (ug/mL) in various organs of *Sparus aurata* fed the experimental diets.

Dietary groups	CTRL	FF-49	FF-97	DF-58	DF-116	DF-174
Foregut	—	—	—	—	—	—
Protein (ug/mL)	55.5 ± 1.2^b^	58.9 ± 2.4^a,b^	54.8 ± 0.4^b^	62.7 ± 1.3^a^	57.7 ± 0.4^a,b^	54.8 ± 3.1^b^
Trypsin (mU/mg)	69.5 ± 7.1	60.1 ± 3.7	66.7 ± 4.7	66.4 ± 7.2	65.6 ± 1.0	63.4 ± 3.3
Alkaline protease (mU/mg)	154.3 ± 15.8	167.0 ± 16.4	169.3 ± 10.9	149.3 ± 5.7	141.7 ± 4.8	151.1 ± 11.2
α-Amylase (mU/mg)	318.6 ± 73.3^c^	384.7 ± 14.6^c^	435.0 ± 21.3^c^	594.6 ± 13.0^b^	671.8 ± 41.5^b^	833.9 ± 4.0^a^
Lipase (mU/mg)	16.2 ± 3.6^d^	39.4 ± 1.2^b^	50.6 ± 2.3^a^	23.0 ± 3.2^c,d^	25.0 ± 1.2^c,d^	31.8 ± 5.3^b,c^
Stomach	—	—	—	—	—	—
Protein (ug/mL)	51.7 ± 0.9^c^	56.8 ± 4.3^a,b^	58.4 ± 0.9^a,b,c^	65.3 ± 2.3^a^	53.4 ± 0.7^c^	63.3 ± 1.3^a,b^
Pepsin (mU/mg)	116.9 ± 19.7	118.0 ± 14.2	108.7 ± 14.7	109.1 ± 6.4	111.0 ± 7.8	119.3 ± 11.6
Maltase (mU/mg)	151.7 ± 50.4	234.2 ± 56.4	251.8 ± 63.1	216.9 ± 56.0	249.5 ± 44.2	252.7 ± 51.5
Aminopeptidase (mU/mg)	79.9 ± 6.3	85.6 ± 8.8	86.6 ± 10.9	78.3 ± 5.3	80.4 ± 9.8	82.3 ± 9.9
Pyloric caeca	—	—	—	—	—	—
Protein (ug/mL)	64.4 ± 1.8^a^	60.5 ± 0.4^b^	54.8 ± 0.6^c^	56.6 ± 0.8^c^	51.2 ± 0.7^d^	64.8 ± 0.4^a^
Trypsin (mU/mg)	90.4 ± 3.7	83.3 ± 2.3	82.8 ± 10.3	85.2 ± 10.0	86.2 ± 2.8	83.8 ± 1.7
Alkaline protease (mU/mg)	176.4 ± 3.3	163.0 ± 6.4	160.1 ± 7.5	180.1 ± 9.7	174.1 ± 1.6	176.7 ± 6.5
α-Amylase (mU/mg)	273.6 ± 19.2	303.8 ± 15.2	324.2 ± 22.3	317.3 ± 28.1	360.4 ± 38.9	406.0 ± 76.7
Lipase (mU/mg)	21.6 ± 3.1^b^	34.1 ± 2.8^b^	48.1 ± 8.9^a^	26.6 ± 0.7^b^	25.0 ± 2.4^b^	32.0 ± 1.1^b^

*Note:* Values represent means ± standard deviation (*n* = 2). Values within each row not sharing a common superscript letter are significantly different (*p*  < 0.05).

**Table 9:** Severity score (0–4) for the observed histomorphological alterations and histological measurements at the foregut of *Sparus aurata* fed the experimental diets.

Dietary groups	CTRL	FF-49	FF-97	DF-58	DF-116	DF-174
Severity score	—	—	—	—	—	—
Liver	1	3	3	1	2	2
Foregut	0	0	0	0	0	0
Histological measurements	—	—	—	—	—	—
Intestinal fold length (mm)	0.41 ± 0.02^b^	0.51 ± 0.02^a,b^	0.49 ± 0.03^a,b^	0.50 ± 0.03^a,b^	0.50 ± 0.02^a,b^	0.54 ± 0.02^a^
Intestinal fold width (mm)	0.13 ± 0.01	0.13 ± 0.01	0.13 ± 0.01	0.14 ± 0.01	0.12 ± 0.01	0.13 ± 0.01
Muscular layer width (mm)	0.06 ± 0.01	0.07 ± 0.02	0.08 ± 0.01	0.08 ± 0.01	0.08 ± 0.01	0.08 ± 0.01
No. goblet cells/intestinal fold	8.00 ± 0.83^a,b^	1.57 ± 0.24^e^	4.22 ± 0.24^c^	9.47 ± 0.67^a^	5.05 ± 0.79^c^	3.83 ± 0.60^c,d^

*Note:* Values represent means ± standard error (*n* = 3). Values within each row not sharing a common superscript letter are significantly different (*p*  < 0.05).

## Data Availability

The data that support the findings of this study are available from the corresponding author upon reasonable request.
